# Complete Genome Sequence of Burkholderia cenocepacia Phage Mica

**DOI:** 10.1128/MRA.01407-20

**Published:** 2021-02-11

**Authors:** James Garcia, Guichun Yao, Maria Guadalupe Vizoso-Pinto, James Clark, Tram Le, Carlos Gonzalez, Jason Gill, Mei Liu

**Affiliations:** a Center for Phage Technology, Texas A&M University, College Station, Texas, USA; b Infection Biology Lab, Instituto Superior de Investigaciones Biológicas, National Council of Scientific and Technological Research, National University of Tucuman, San Miguel de Tucumán, Argentina; Loyola University Chicago

## Abstract

Burkholderia cenocepacia is a multidrug-resistant Gram-negative pathogen known to colonize patients with chronic granulomatous disease and cystic fibrosis. Here, we describe *Burkholderia* phage Mica, which is predicted to be a lysogenic myophage based on the similarity of its structural proteins to *Enterobacteria* phage P2 and *Burkholderia* phage KL3.

## ANNOUNCEMENT

Bacteria within the genus *Burkholderia* are known to cause disease in plants. Burkholderia cenocepacia is also a multidrug-resistant Gram-negative bacterium within the B. cepacia complex (BCC) group of opportunistic pathogens that colonizes patients with cystic fibrosis and chronic granulomatous disease (1, 2). The investigation into *Burkholderia* phage may yield an effective biocontrol agent that can aid in the treatment of afflicted patients as well as protect crop plants.

Phage Mica was isolated from a soil sample collected from Hermann Park in Houston, TX (GPS coordinates, 29.7135373, −95.3910571). The filtered soil extract (soil sample mixed with phosphate-buffered saline [PBS] buffer) was enriched against a B. cenocepacia clinical strain (MS1) in tryptic nutrient broth at 37°C overnight. Phage was purified and propagated from the enrichment using the soft-agar overlay method (3) on B. cenocepacia strain MS1 grown on tryptic nutrient agar at 37°C. Genomic DNA was purified by a modification of the Promega Wizard DNA kit protocol described by Summer (4). DNA libraries were prepared with 550-bp inserts using an Illumina TruSeq Nano kit. The paired-end 500-bp reads were sequenced on an Illumina MiSeq instrument using v2 500-cycle chemistry. The 307,034 total sequence reads were quality controlled with FastQC v0.11.9 (www.bioinformatics.babraham.ac.uk/projects/fastqc) and trimmed with the FASTX-Toolkit v0.0.14 (http://hannonlab.cshl.edu/fastx_toolkit/). The genome sequence was assembled with SPAdes v3.5.0 with default parameters (5). A single contig with 200.1-fold coverage was confirmed to be closed with Sanger sequencing that was PCR amplified by primers (5′-TCGAACGACCATTCGATGC-3′ and 5′-ACATTGCCAGCCTGCAGG-3′). Protein-coding genes were called by GLIMMER v3.0 and MetaGeneAnnotator v1.0 (6, 7). tRNA genes were determined by ARAGORN v2.36, and rho-independent terminators were predicted with TransTermHP v2.09 (8, 9). Gene functions were predicted by conserved domains identified with InterProScan v5.33 and similarities found using BLASTp v2.9.0 with a 0.001 maximum expectation value cutoff against the NCBI nonredundant (nr) and Swiss-Prot databases (10–12). The prediction of transmembrane domains was performed with TMHMM v2.0 (13). The prediction of spanin proteins was performed based on the analysis of lipoprotein signals with LipoP v1.0 (14). Further domain analysis was performed with HHpred v3.2.0 (15). Genome-wide DNA sequence similarity was calculated by progressiveMauve v2.4 (16). All annotation tools accessed through the CPT Galaxy and Web Apollo interfaces (17–19) were run at default settings unless otherwise stated.

Mica has a genome sequence of 43,707 bp with a coding density of 94% and a G+C content of 62%. A cohesive end site (*cos*) with a 13-bp overhang was predicted by PhageTerm (20). Analysis predicted 69 protein-coding genes, of which 43 were assigned putative functions. Mica was predicted to be a lysogenic myophage based on presence of an integrase and the similarity of its structural proteins to phage P2 (GenBank accession no. NC_001895) and *Burkholderia* phage KL3 (NC_015266). BLASTn indicated that phage Mica shares 47% similarity to myophage phiRSP (MH252365). The lysis cassette genes were found in a cluster and consist of i/o-spanins, a signal anchor release (SAR) endolysin lysozyme, and a class II holin. An ice nucleation protein is predicted through functional analysis, which may act as an accessory phytopathogen virulence factor in the lysogen (21).

### Data availability.

The genome sequence of phage Mica was deposited under GenBank accession no. MT701586 and BioSample accession no. SAMN14609637. The BioProject accession no. is PRJNA222858, and the SRA accession no. is SRR11558335.
